# LKB1 pro‐oncogenic activity triggers cell survival in circulating tumor cells

**DOI:** 10.1002/1878-0261.12111

**Published:** 2017-09-30

**Authors:** Elisabeth Katharina Trapp, Leonie Majunke, Beate Zill, Harald Sommer, Ulrich Andergassen, Julian Koch, Nadia Harbeck, Sven Mahner, Thomas Wolfram Paul Friedl, Wolfgang Janni, Brigitte Rack, Marianna Alunni‐Fabbroni

**Affiliations:** ^1^ Department of Gynecology and Obstetrics Breast Center Ludwig‐Maximilians‐University Munich Germany; ^2^ Department of Gynecology and Obstetrics University Hospital Ulm Germany

**Keywords:** anoikis, circulating tumor cell, epithelial‐to‐mesenchymal transition, metabolic stress, metastatic breast cancer, tumor suppressor liver kinase B1

## Abstract

During intravasation, circulating tumor cells (CTCs) detach from the epithelium of origin and begin the epithelial‐to‐mesenchymal transition (EMT) process, where they lose epithelial features and pass through the endothelium to enter circulation. Although detachment from the extracellular matrix is a strong source of metabolic stress, which induces anoikis, CTCs can survive. Recently, the tumor suppressor liver kinase B1 (LKB1) has gained attention for its role as a proto‐oncogene in restoring the correct ATP/AMP ratio during metabolic stress. The aim of this study was to assess LKB1 expression in epithelial‐negative CTCs isolated from patients with metastatic breast cancer and to characterize its possible association with EMT and stemness features. Transcriptome analysis of EpCAM‐negative CTCs indicated that over 25% of patients showed enhanced LKB1 levels, while almost 20% of patients showed enhanced levels of an EMT transcription factor known as ZEB1. Transcriptome and immunofluorescence analyses showed that patients with enhanced LKB1 were correspondingly ZEB1 negative, suggesting complementary activity for the two proteins. Only ZEB1 was significantly associated with cancer stem cell (CSC) markers. Neither LKB1 nor ZEB1 upregulation showed a correlation with clinical outcome, while enhanced levels of stemness‐associated CD44 correlated with a lower progression‐free and overall survival. *Ex vivo* models showed that MDA‐MB‐231, a mesenchymal tumor cell line, grew in suspension only if LKB1 was upregulated, but the MCF‐7 epithelial cell line lost its ability to generate spheroids and colonies when LKB1 was inhibited, supporting the idea that LKB1 might be necessary for CTCs to overcome the absence of the extracellular matrix during the early phases of intravasation. If these preliminary results are confirmed, LKB1 will become a novel therapeutic target for eradicating metastasis‐initiating CTCs from patients with primary breast cancer.

**Abbreviations**
CEC: circulating endothelial cell
CSC: cancer stem cell
CTC: circulating tumor cell
ECM: extracellular matrix
EMT: epithelial‐to‐mesenchymal transition
EpCAM: epithelial cell adhesion molecule
LKB1: tumor suppressor liver kinase B1
MET: mesenchymal–epithelial transition
TIC: tumor‐initiating cell
ZEB1: zinc‐finger E‐box‐binding homeobox factor 1

## 1 Introduction

Despite significant progress toward achieving a high 5‐year survival rate in the treatment for breast cancer (BC), some patients, nevertheless, relapse and die from incurable metastatic disease (Agopian *et al*., 2017; Cote *et al*., 1991; Diel *et al*., 1996; Jung *et al*., 2012). Over the last decade, the role of epithelial circulating tumor cells (CTCs) in metastatic induction and their prognostic power has been thoroughly demonstrated (Cristofanilli *et al*., 2004, 2005; Hayes *et al*., 2006; Janni *et al*., 2016; Rack *et al*., 2014; Riethdorf *et al*., 2007; Wallwiener *et al*., 2013). To disseminate and form distal metastasis, CTCs must leave the epithelium of origin, cross the endothelium (intravasation), enter circulation (where they must survive shear stress, an absent extracellular matrix (ECM), and the immune system response), exit the circulation (extravasation), and attach to the new tissue (Bednarz‐Knoll *et al*., 2012). To intravasate, tumor cells undergo the epithelial‐to‐mesenchymal transition (EMT), where they lose the EpCAM‐positive (EpCAM^+^) epithelial phenotype, acquiring invasive properties (Giordano *et al*., 2012; Pantel and Speicher, 2016). EMT is linked to several cancerogenesis hallmarks, changes in adhesion and motility properties, and the acquisition of cancer stem cell (CSC) characteristics (Hanahan and Weinberg, 2000; Kasimir‐Bauer *et al*., 2012; Mani *et al*., 2008) and is a major driving force for tumor progression, metastasis induction, chemotherapeutics resistance, and poor prognosis (Fischer *et al*., 2015; Soltermann *et al*., 2008). EMT is tightly regulated by expression changes in several genes. For example, the cadherin switch, with its upregulation of the mesenchymal N‐cadherin and downregulation of the epithelial E‐cadherin, enables detachment from neighboring epithelial cells. A key player in this process is the zinc‐finger E‐box‐binding homeobox factor 1 (ZEB1), a transcription factor whose aberrant upregulation in tumor cells is directly linked to loss of E‐cadherin, reduced cellular adhesion, increased invasiveness, changes in cell polarity, and metastasis development (Karihtala *et al*., 2013; Spaderna *et al*., 2008; Zhang *et al*., 2015). In CSC subpopulations of various tumors, ZEB1 participates in a positive feedback loop with CD44 (Li *et al*., 2007; Patrawala *et al*., 2006; Preca *et al*., 2015) and ZEB1 overexpression is evident in CTCs that display a CSC‐ phenotype (Giordano *et al*., 2012). In concordance with these findings, in gastric cancer, CD44 is also a marker of a phenotypic subgroup of CTCs known as tumor‐initiating cells (TICs), conferring a strong ability in these cells to induce disease recurrence and metastasis (Li *et al*., 2014b). In this respect, ZEB1 may also be considered as a hallmark of TICs. During intravasation, CTCs face different physiological hurdles. Specifically, loss of adhesion to ECM leads to anoikis, a form of apoptosis that is induced by detachment from a solid surface. To survive, CTCs must activate mechanisms to block cell death. Several oncogenes and pathways have been proposed in cell death rescue (Frisch and Screaton, 2001). Recently, the tumor suppressor liver kinase B1 (LKB1), also known as serine/threonine kinase 11 (STK11), has gained attention for its dual role as a tumor suppressor and proto‐oncogene (Ollila and Makela, 2011). LKB1 displays tumor‐suppressing characteristics; its upregulation inhibits metastasis (Zhuang *et al*., 2006), while its downregulation or deletion promotes EMT and cancer progression (Li *et al*., 2014a). Moreover, a correlation between LKB1 downregulation and ZEB1 upregulation with concomitant induction of EMT in tumor cells has been described (Yao *et al*., 2016). However, LKB1 is also a potent sensor of metabolic low energy. In response to cellular stress (i.e., low levels of glucose and hypoxia), ATP levels fall and AMP levels rise with the induction of a strong signal for cellular death. LKB1 is essential for restoring the correct ratio between ATP and AMP via AMPK to prevent the initiation of apoptosis. In this context, LKB1 functions as a proto‐oncogene (Green *et al*., 2011; Hardie *et al*., 2012; Lee *et al*., 2015; Peart *et al*., 2015; Shaw *et al*., 2004). The aim of our study was to investigate EpCAM‐negative CTCs that were isolated from patients with metastatic breast cancer (mBC) and focus on LKB1 and ZEB1 expression to dissect their functional roles in triggering anoikis and tumor progression. Understanding the mechanisms that allow tumor cells to grow in the absence of a substrate and acquire metastatic potential may offer new strategies for earlier and more effective therapeutic approaches in medical oncology.

## 2 Materials and methods

### 2.1 Ethics approval

Blood samples were collected from patients with mBC, who were recruited between October 2013 and April 2016 in the Department of Gynecology and Obstetrics, Ludwig‐Maximilians‐University (Munich, Germany). The study was approved by the institutional ethical board and conducted in accordance with the Declaration of Helsinki (World Medical, 2013). All participants provided informed consent.

### 2.2 Patients’ characteristics

Twenty‐seven patients with mBC were included in this pilot study. All patients [mean age (years ± SD): 58.52 ± 12.50, range 35–77] had histologically confirmed primary BC per standard clinical guidelines. Tumor classification was performed according to the TNM guidelines (Singletary *et al*., 2002). Luminal cancer type A was defined as estrogen and/or progesterone receptor‐positive (ER^+^/PR^+^), human epidermal growth factor receptor 2‐negative (HER2^−^) and grade (G) 1–2; luminal cancer type B was defined as ER^+^/PR^+^, HER2‐ positive (HER2^+^) or HER2^−^ and G3; basal‐like tumor was defined as ER^−^/PR^−^ and HER2^−^ (triple negative, TN); and the HER2‐like tumor was defined as HER2^+^. The HER2 status was assessed at the Pathology Department (Ludwig‐Maximilians‐University) according to the standard protocols. Patients’ clinical and histopathological details are summarized in Table 1. Seventeen female healthy donors (HDs) (mean age, years ± SD: 51 ± 9.7, range: 34–63) with no history of cancer were included in the study as negative controls.

**Table 1:** Patients’ and primary tumor characteristics. (ER, estrogen receptor; PR, progesterone receptor; na, not available)

Total	27^a^
Mean age (±SD)	58.5 ± 12.5
Range (years)	35–77
Tumor type (%)	
Ductal	24 (88.9)
Lobular	3 (11.1)
Tumor size (pT) (%)	
pT1a‐c	4 (14.8)
pT2‐4	23 (85.2)
Lymph node status (pN) (%)	
Negative (pN0)	9 (33.3)
Positive (pN1‐3)	14 (51.9)
na	4 (14.8)
Grading (G) (%)	
G1‐2	10 (37.0)
G3	14 (51.9)
na	3 (11.1)
Hormone receptor status (ER/PR) (%)	
Negative	5 (18.5)
Positive	22 (81.5)
HER2 status (%)	
Negative	21 (77.8)
Positive	6 (22.2)
Menopausal status (%)	
Premenopausal	14 (51.9)
Postmenopausal	13 (48.1)
Primary surgery (%)	
Breast conservative	13 (48.1)
Mastectomy	6 (22.2)
Both	3 (11.1)
None	5 (18.6)
Systemic therapy (%)	
Chemotherapy	12 (44.4)
Endocrine	21 (77.7)
Herceptin	6 (22.2)
Radiotherapy	16 (59.9)

a Number of patients (percentage).

### 2.3 Enrichment of EpCAM‐negative CTCs by immunodepletion

Peripheral blood (PB) (5 mL) from patients and HDs was directly drawn in EDTA tubes (Becton Dickinson, Heidelberg, Germany), immediately stored at 4 °C, and processed within 4 h of collection. To deplete the blood samples from EpCAM^+^ CTCs, the AdnaTest BreastCancerSelect Kit (Qiagen Hannover GmbH, Langenhagen, Germany) was used according to the manufacturer's instructions (for details, see Appendix S1). Immunodepleted cell pellets were resuspended in phosphate**‐**buffered saline (pH 7.2) (PBS) (5 mL) and centrifuged at 300 ***g*** for 10 min at room temperature (RT). A total of 1 × 10^7^ cells were resuspended in 80 μL of PBE buffer containing PBS, 0.5% bovine serum albumin, and 2 mm EDTA, mixed with 20 μL of CD45 MicroBeads (Miltenyi Biotec, Bergisch Gladbach, Germany), incubated at 4 °C for 15 min, washed in PBE (2 mL), and centrifuged at 300 ***g*** for 10 min at RT. After removal of the supernatant, cells were resuspended in PBE (500 μL). Before processing the magnetic separation with MACS LS columns (Miltenyi Biotec) and the quadroMACS separator (Miltenyi Biotec), the columns were placed into the magnetic separator and activated by rinsing with PBE (3 mL). After applying the cell suspension to the column, the eluate was collected. The column was washed three times with PBE (3 mL) for each washing step and all eluates were collected. Cells were pelleted by centrifugation at 300 ***g*** for 10 min at RT, supernatants were removed, and pellets were stored at −20 °C until further use. Unfortunately, with the type of cellular selection we performed, we cannot completely exclude the expression of the transcripts also by other cells types with a EpCAM^−^/CD45^−^ phenotype but lacking a tumoral origin, such as circulating endothelial cells.

### 2.4 Isolation of total RNA

Total RNA isolation was performed using the TRIzol LS Reagent (ThermoFisher Scientific, Darmstadt, Germany) according to the manufacturer's instructions (for details, see Supplemental Experimental Materials). DNase‐treated samples were reverse‐transcribed using the SuperScript III First‐Strand Synthesis SuperMix (ThermoFisher Scientific) according to the manufacturer's instructions. In the RT‐negative controls, RT enzyme was replaced by DNase/RNase‐free water. cDNA was stored at −20 °C until use.

### 2.5 Quantitative real‐time PCR

Quantitative real‐time PCR (qPCR) was performed using a final reaction mix volume of 20 μL, which contained cDNA (2 μL), 20X TaqMan Gene Expression Assay reagent (ThermoFisher Scientific) (1 μL), 2X TaqMan Fast Universal PCR Master Mix no AmpErase UNG (10 μL) (ThermoFisher Scientific), and RNase/DNase‐free water (7 μL). The complete list of hydrolysis probes used in this study is presented in Table S1 (for details, see Supplemental Experimental Materials). All samples were run in duplicate, and no‐template controls were included on each plate for all assays. The plate was loaded into the 7500 Fast Real‐Time PCR system (ThermoFisher Scientific) using the amplification standard mode (50 °C for 2 min, 95 °C for 10 min and 40 cycles at 95 °C for 15 s and 60 °C for 60 s). Relative mRNA expression was calculated using the equation 2^−ΔCq^, where ΔCq = (Cq target mRNA)−(Cq reference mRNA) (Livak and Schmittgen, 2001). The equation 2^−ΔΔCq^ was used to calculate the fold difference in mRNA between patients with mBC and HDs, using ΔΔCq = [(Cq target mRNA)−(Cq reference mRNA)]_patients_−[(Cq target mRNA)−(Cq reference mRNA)]_HD_ (Livak and Schmittgen, 2001). Each primer was separately tested to define the PCR amplification efficiency using calibration curves. The correlation coefficient (*R* ^2^) and PCR efficiency, which were calculated from the slope, fell between 0.988 and 0.999 and 82.9% and 104.8%, respectively (for details, see Appendix S1 – Table S1). Comparing 10 different reference genes with the Normfinder algorithm, β‐actin was identified as the best reference gene to normalize Cq values (Andersen *et al*., 2004). A gene was considered highly expressed if the relative 2^−ΔCq^ value was higher than the mean 2^−ΔCq^ + 1 standard deviation (SD) in HDs (Giordano *et al*., 2012).

### 2.6 Quantification of EpCAM^+^ CTCs

EpCAM^+^ CTCs were enriched and quantified using the CellSearch™ System (Janssen Diagnostics, LLC, Raritan, NJ, USA). Peripheral blood was drawn into CellSave tubes (10 mL) (Janssen Diagnostics) and analyzed within 24 h according to the manufacturer's recommendations (for details, see Supplemental Experimental Materials). CTCs were defined as nucleated cells lacking CD45 and expressing cytokeratin. Patients presenting at least one CTC were defined as CellSearch positive (CS^+^); all the others were CellSearch negative (CS^−^).

### 2.7 Cell lines and tumor sphere culture

The tumor breast cancer cell lines MCF‐7 and MDA‐MB‐213 were obtained from the European Collection of Authenticated Cell Cultures (ECACC, England, UK) and cultured in high glucose Dulbecco's modified Eagle medium (DMEM) (Biochrom GmbH, Berlin, Germany) with 10% fetal bovine serum (Biochrom GmbH), 100 μg·mL^−1^ penicillin/streptomycin (Biochrom GmbH), and 2.5 μg·mL^−1^ amphotericin (Biochrom GmbH) at 37 °C in a humidified 5% CO_2_ incubator. For tumor sphere formation, cells were seeded into ultra‐low adhesion (ULA) plates (Corning Inc., Corning, NY, USA) at 1 × 10^3^ cells/6‐cm plate in serum‐free DMEM/F12 (Biochrom GmbH) supplemented with 100 μg·mL^−1^ penicillin/streptomycin (Biochrom GmbH), 2.5 μg·mL^−1^ amphotericin (Biochrom GmbH), 10 ng·mL^−1^ human recombinant epidermal growth factor (hrEGF) (Sigma‐Aldrich Chemie GmbH, Munich, Germany), 20 ng·mL^−1^ human recombinant basic fibroblast growth factor (hrbFGF) (Sigma‐Aldrich Chemie GmbH), and 2% Gibco‐B27 supplement (ThermoFisher Scientific). Cells remained in culture for 7 days. Tumor spheres were either collected as individual spheres and transferred onto glass slides (SuperFrost^®^Plus, ThermoFisher Scientific) for immunostaining and to 0.2 mL PCR tubes for transcriptome analysis, or pooled by centrifugation (300 ***g*** for 5 min), after which they were mechanically dissociated through a 23‐G needle, resuspended in serum‐free DMEM/F12 medium, and plated to ULA 6‐well plates to reform spheres. The same procedure was followed 7 days later. After 21 days, spheres with minimal diameters of 20 μm were counted and transferred to glass slides for immunostaining or to 0.2‐mL PCR tubes for transcriptome analysis.

### 2.8 Small interfering RNA (siRNA) transfections

MCF‐7 cells (2.5 × 10^5^ cells per well) were seeded into 6‐well plates (Corning Inc.) at 60% confluence in DMEM containing 10% serum. After 24 h, cells were transfected with siRNAs (100 nm) using the Lipofectamine RNAiMAX Reagent (ThermoFisher Scientific) according to manufacturer's recommendations. The LKB1‐specific siRNA (sc‐35816) and the control nonspecific (NS)‐siRNA (sc‐36869) were purchased from Santa Cruz Biotechnologies (Santa Cruz, CA, USA). Cells were harvested 48 h after transfection and further analyzed for tumor sphere formation or clonogenic assay. RNA extracted from transfected or untransfected (mock) MCF‐7 cells was used to monitor the transfection efficiency by RT‐qPCR. Five independent transfection experiments were performed, and each analysis was performed in triplicate. Transfection efficiency was calculated with the two‐tailed Friedman's test (*P *=* *0.0015).

### 2.9 Clonogenic assay

Transfected or mock MCF‐7 cells were trypsinized, counted, and seeded (1 × 10^3^/dish) in triplicate into 12‐cm dishes (Corning Inc.). They were allowed to form colonies for 14 days and stained with crystal violet (0.05%) in 3.7% formaldehyde (1%) and methanol (1%). Colonies with at least 50 cells were counted.

### 2.10 Tumor sphere qPCR analysis

Single tumor spheres were collected and transferred to 0.2‐mL PCR tubes that were filled with PBS (30 μL) and centrifuged at 800 ***g*** for 2 min at RT. The supernatant was discarded, and the pellet was lysed in 5 μL of CelluLyser buffer (TATAA Cellulyse MicroLysis, TATAA Biocenter, Göteborg, Sweden) mixed with DNase/RNase‐free water (4 μL), and Universal RNA Spike I (1 μL) (TATAA Biocenter). The lysed cells were immediately transferred to two fresh tubes (5 μL each) and mixed with RT‐master mix (5 μL) which included DNase/RNase‐free water (2.5 μL), TATAA GrandScript Reaction Mix (2 μL) (TATAA Biocenter), and TATAA GrandScript RT Enzyme (0.5 μL) (TATAA Biocenter). The RT enzyme was substituted with the same volume of water in RT‐negative controls. cDNA synthesis was performed by incubating the samples at 22 °C for 5 min, at 42 °C for 30 min, and at 85 °C for 5 min. The cDNA was diluted with 130 μL of water and 2 μL was transferred to a fresh tube for qPCR analysis as previously described.

### 2.11 Cytospins preparation

Whole blood from patients and HDs was collected in 10‐mL EDTA vacutainer tubes (Becton Dickinson), and cytospins were prepared as previously described (Rack *et al*., 2016) (for details, see Appendix S1).

### 2.12 Immunofluorescence analysis

Cytospins or slides with single tumor spheres were thawed at room temperature, permeabilized for 5 min in cold (−20 °C) methanol (Merck, Darmstadt, Germany), dried for 15 min at RT, washed three times for 5 min in PBS, and incubated for 20 min in Ultra Vision Protein Blocking medium (ThermoFisher Scientific) to reduce background staining. Cytospins were first incubated for 60 min at RT with a rabbit polyclonal anti‐human ZEB1 antibody (clone H‐102; Santa Cruz Biotechnology, dilution 1 : 1000), washed three times for 5 min with PBS, and incubated for 45 min at RT with the Cy3‐conjugated AffinityPure Fab Fragment goat anti‐rabbit IgG (H+L) (Jackson ImmunoResearch Laboratories Inc., West Grove, PA, USA, dilution 1 : 200). Cytospins were washed two times for 5 min with PBS and incubated either for 60 min at RT with mouse monoclonal anti‐human CD44 (clone DF‐1485, Santa Cruz Biotechnology, dilution 1 : 200) or for 4 h at 37 °C with mouse monoclonal anti‐human LKB1 (clone E‐9, Santa Cruz Biotechnology, dilution 1 : 50), washed three times for 5 min with PBS, and incubated for 45 min with the Cy2‐conjugated AffinityPure Fab Fragment goat anti‐mouse IgG (H+L) (Jackson ImmunoResearch Laboratories Inc., dilution 1 : 200). After two 5‐min washing steps in PBS, slides were mounted with ProLong Diamond Antifade Mountant with 4′6‐diamidino‐2‐phenylindole (DAPI) (ThermoFisher Scientific) and analyzed with a fluorescence microscope (Axiophot, Carl Zeiss, Germany) using a 20‐ or 40‐fold magnification. Samples that were only stained with the secondary antibodies were used as negative controls to assess nonspecific staining.

### 2.13 Statistical analysis

Statistical analysis was performed using graphpad prism version 6.00 (GraphPad Software, La Jolla, CA, USA). The nonparametric two‐tailed Mann–Whitney test was used to compare transcripts’ levels between patients and HDs or between different cell lines and derived tumor spheres. The association between mRNA levels and the patient’ clinical characteristics was evaluated using the chi‐square test or Fisher's exact test (if the expected frequency in at least one cell of a 2 × 2 table was less than 5). The transfection efficiency or the differences between LKB1‐siRNA‐treated cells, NS‐siRNA‐treated cells, and mock controls were assessed for statistical significance with the two‐tailed Friedman's test. Overall survival (OS) and progression‐free survival (PFS) were analyzed using the Kaplan–Meier method, and survival estimates in different groups were compared using the log‐rank test. OS was calculated from the date of primary tumor diagnosis to the date of death or the date of the last clinical follow‐up. PFS was calculated from the date of primary tumor diagnosis to the date of first metastasis detection. Two‐sided *P*‐values below 0.05 were considered statistically significant. No adjustment of the significance level for multiple testing was performed.

## 3 Results

### 3.1 Patient characteristics

The study population included 27 patients with mBC and 17 HDs. Patients relapsed after several years from primary diagnosis and started a new treatment line or had a documented progressive BC before receiving a new therapy. The mean patient age was 58.5 ± 12.5 years (range, 35–77); almost half were postmenopausal (*n* = 13, 48.1%). Most presented with ductal primary BC (*n* = 24, 88.9%), received a breast conservative surgical treatment (*n* = 13, 48.1%), and different systemic treatment types (alone or in combination), including chemotherapy (*n* = 12, 44.4%), endocrine therapy (*n* = 21, 77.7%), HER2‐directed therapy (herceptin) (*n* = 6, 22.2%), and radiotherapy (*n* = 16, 59.2%). Most patients presented with hormone receptor‐positive (*n* = 22, 81.5%) and HER2‐negative (*n* = 21, 77.8%) primary tumors; in most cases, the tumor stage was pT2‐4 (*n* = 23, 85.2%), the nodal status was pN1‐3 (*n* = 14, 51.9), and the grade was G3 (*n* = 14, 51.9%). Patient details and primary tumor characteristics are presented in Table 1.

### 3.2 Transcriptome characterization of EpCAM^**−**^ CTCs

**Figure 1:**
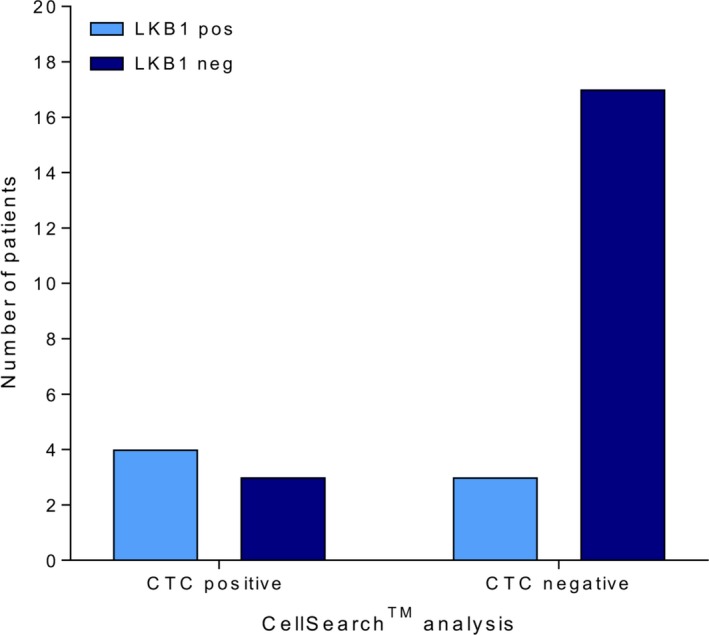
Association between the presence of EpCAM ^+^ CTCs in CS ^+^ patients and the detection of LKB1 in the immunodepleted blood fraction. LKB1 RNA was quantified by RT‐qPCR. Samples were considered positive if gene expression by 2^−ΔCq^ was equal to or higher than 2^−ΔCq^ + 1 SD as found in healthy donors. Association was calculated using Fisher's exact test (*P *=* *0.049).

EpCAM/CD45‐immunodepleted blood fractions were first analyzed with respect to the expression of different genes that defined the characteristics of EpCAM^−^ CTCs. We observed that 37% of the patients (*n* = 10) showed enhanced levels of the mesenchymal marker vimentin, only 7.4% of the patients (*n* = 2) showed enhanced levels of the epithelial marker E‐cadherin, and 25.9% (*n* = 7) of the patients showed enhanced levels of at least one transcription factor (TF) linked to EMT among SLUG, ZEB1, SNAIL1, and Twist 1, with ZEB1 being the most frequent (18.5%, *n* = 5). LKB1 was upregulated in 25.9% (*n* = 7) of the samples analyzed and none of the patients who were positive for LKB1 were positive for ZEB1. Furthermore, we detected enhanced levels of at least one cancer stem cell (CSC) marker among CD133, CD24, and CD44 in 59% of the samples analyzed (*n* = 14) (Table 2). The same analysis was performed after grouping the patients according to the CellSearch™ analysis, which scored 26% (*n* = 7) of overall patients as CTC‐positive (CS^+^) and 74% (*n* = 20) as CTC‐negative (CS^−^). When we analyzed the immunodepleted blood fractions and compared the results between the CS^+^ and CS^−^ subgroups, we observed that CS^−^ patients were more often positive for CSC markers (for CD24: 40%, *n* = 8 vs. 33.3%, *n* = 2; for CD44: 40%, *n* = 8 vs. 16.6%, *n* = 1; for CD133: 15%, *n* = 3 vs. 0%, *n* = 0), for vimentin (40%, *n* = 8 vs. 28.5%, *n* = 2), for E‐cadherin (10%, *n* = 2 vs. 0%, *n* = 0), and for ZEB1 (20%, *n* = 4 vs. 14.2%, *n* = 1). Conversely, compared to CS^+^ patients, the CS^−^ patients were less often positive for LKB1 (15%, *n* = 3 vs. 57.1%, *n* = 4) (Table 2). None of the markers analyzed could differentiate the two groups (Mann–Whitney U‐test, all *P > *0.05). However, a positive association between the enhanced levels of LKB1 in the immunodepleted cell fraction and the presence of EpCAM^+^ CTCs in CS^+^ patients was observed (Fisher's exact test, *P = *0.049, concordance rate 77.7%) (Fig. 1).

**Table 2:** Gene expression analysis in immunodepleted blood fractions isolated from patients with mBC. A panel of different RNA transcripts corresponding to different cellular markers was tested by RT‐qPCR in immunodepleted blood fractions. Samples were considered positive if gene expression by 2^−ΔCq^ was equal to or higher than 2^−ΔCq^ + 1 SD as found in healthy donors. The same type of analysis was performed in the whole cohort (All) or in the cohort that was grouped according to the positivity (CS^+^) or negativity (CS^−^) of the patients in the CellSearch™ (CS) analysis. Stem cell marker (SCM); CellSearch™ (CS); (*n = 26) (**n = 6)

	All (*n* = 27)	CS^+^ only (*n* = 7)	CS^−^ only (*n* = 20)
Vimentin	10 (37.0%)	2 (28.5%)	8 (40.0%)
E‐Cadherin	2 (7.4%)	0 (0.0%)	2 (10.0%)
ZEB1	5 (18.5%)	1 (14.2%)	4 (20.0%)
LKB1	7 (25.9%)	4 (57.1%)	3 (15.0%)
CD24	10* (38.4%)	2** (33.3%)	8 (40.0%)
CD44	9* (34.6%)	1 (16.6%)	8 (40.0%)
CD133	3* (11.5%)	0 (0.0%)	3 (15.0%)
All SCM	14* (59.0%)	2 (7.7%)	13 (50.0%)

### 3.3 Relative quantification of transcripts in CS^+^ and CS^−^ patients

**Figure 2:**
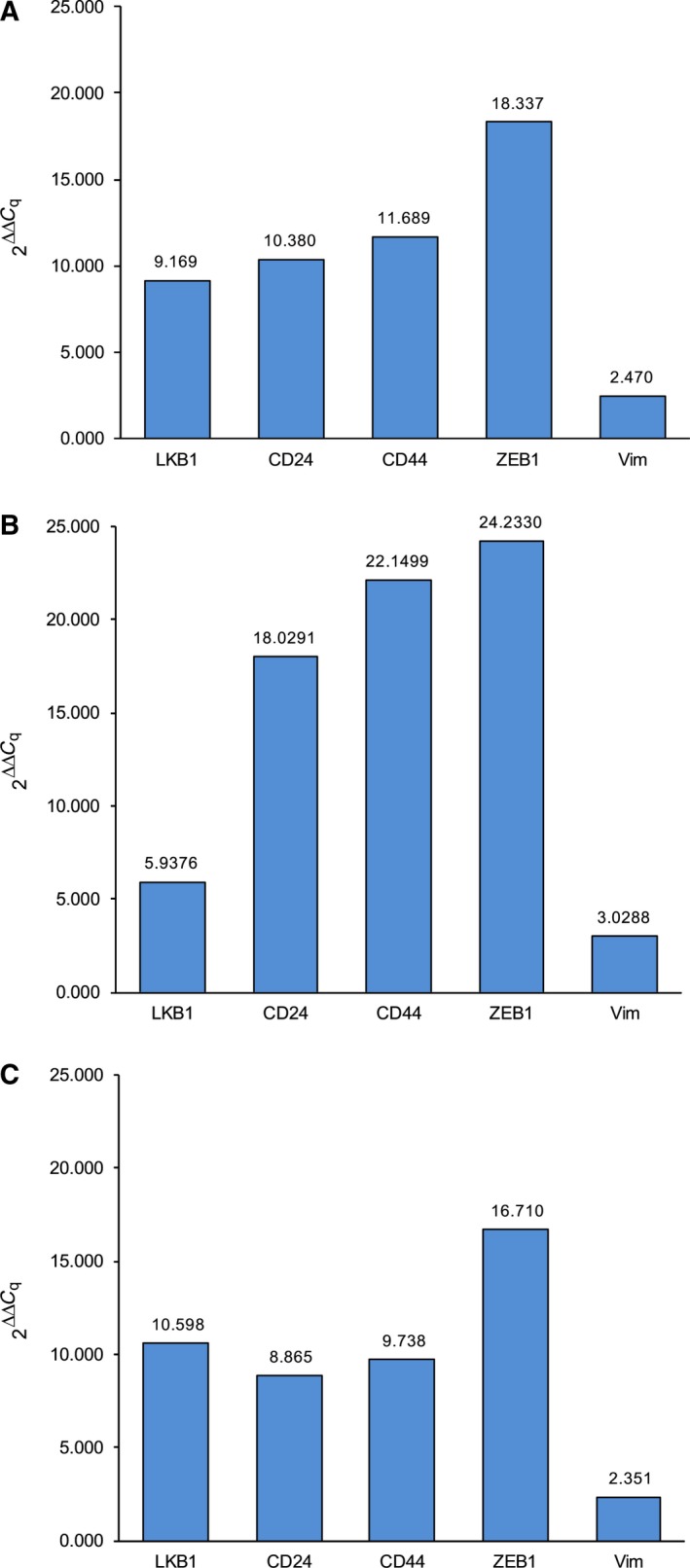
Relative gene expression level in the immunodepleted fraction of all patients (panel A), CS ^+^ patients only (panel B), or CS ^−^ patients only (panel C). β‐Actin served as the reference gene for normalization, and the expression of each gene relative to healthy donors was calculated using the equation 2^−ΔΔCq^.

The relative levels of the most frequently detected transcripts were quantified in the three groups of patients (all, CS^+^, and CS^−^). The mean fold changes in the ZEB1, CD24, CD44, LKB1, and vimentin transcripts were measured using the ΔΔCq method to calculate the relative quantity (RQ). Comparisons of the values for each immunodepleted fraction in all patients relative to HDs showed that the ZEB1 levels were almost 20 times higher; CD24, CD44, and LKB1 levels were increased by approximately 10‐fold, while vimentin was only moderately higher (Fig. 2A). When we grouped the patients according to CellSearch™ and compared the corresponding RQs values, we observed that the relative transcript levels for ZEB1, CD24, and CD44 were enhanced in the CS^+^ group compared to the CS^−^ group, while the LKB1 level almost doubled in the CS^−^ group compared to the CS^+^ group (Fig. 2B,C).

### 3.4 Association between ZEB1 and LKB1 expression and other cellular markers, clinicopathological characteristics, and clinical outcome

**Figure 3:**
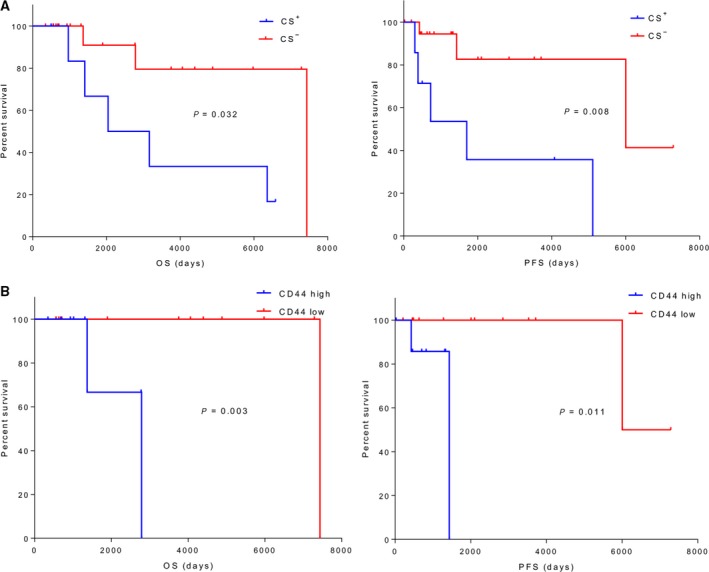
Kaplan–Meier plots for overall survival (OS) and progression‐free survival (PFS) in patients with mBC. (A) EpCAM ^+^ CTC detection in CS ^+^ patients was associated with a lower OS (*P *=* *0.032) and a lower PFS (*P *=* *0.008) relative to CS ^−^ patients. (B) Enhanced levels of CD44 in the immunodepleted fraction of CS ^−^ patients were associated with a lower OS (*P *=* *0.003) and a lower PFS (*P *=* *0.011) relative to patients with baseline levels of CD44. Samples were considered positive for CD44 if gene expression by 2^−ΔCq^ was equal to or higher than 2^−ΔCq^ + 1 SD as found in HDs. OS and PFS were analyzed using the Kaplan–Meier method, and survival estimates in different groups were compared using the log‐rank test.

Within the patient cohort, we observed that ZEB1 was the most often upregulated EMT‐TF marker, with 20% of the CS^−^ patients showing enhanced ZEB1 levels in their immunodepleted blood fractions. Enhanced ZEB1 expression was weakly associated with CD24 expression (*P = *0.054; concordance rate 73.0%). Conversely, ZEB1 expression was not associated with the expression of different phenotypic markers, such as vimentin (*P *=* *0.326), E‐cadherin (*P *=* *1.000), plastin 3 (*P *=* *1.000), or LKB1 (*P *=* *0.283) (Table 3). The expression level of ZEB1 was also analyzed with respect to the clinicopathological characteristics of the patients at the time of the primary diagnosis. We observed a significant association between the ZEB1 level and the grade of differentiation shown by the original primary tumor (*P = *0.020, concordance rate 75%). We did not observe associations between ZEB1 and other clinical parameters such as tumor stage, nodal stage, HER2 status, hormone receptor status, histological type, localization of metastasis, or therapies (chemo‐, hormone‐, radio‐, or herceptin therapy) (all *P *>* *0.1) (Table 4). Similarly, the association between enhanced expression levels of LKB1, the cellular markers, and the clinicopathological characteristics was evaluated. The expression of LKB1 was not significantly associated with ZEB1, CD24, CD44, E‐cadherin, or vimentin or with any of the clinicopathological factors investigated (all *P > *0.1; see Appendix S1 – Table S2). The relative expression of the different cellular markers was evaluated also with respect to the clinical outcome. Patients first diagnosed with BC without any sign of metastasis (*n* = 24) showed disease progression after a median of 37 months from the primary diagnosis. Of this group, eight patients died at a median time of 61 months after the first diagnosis. As expected, the CS^+^ patients had a lower overall survival (OS) and progression‐free survival (PFS) than the CS^−^ patients (*P *=* *0.032 and *P *=* *0.008, respectively) (Fig. 3A). Of the CS^−^ patients, only those with enhanced CD44 levels showed a significantly lower OS and PFS (*P *=* *0.003 and *P *=* *0.011, respectively) (Fig. 3B). On the contrary, we did not observe significant associations between OS/PFS and LKB1 or other markers (all *P* > 0.05). However, due to the relatively small cohort size, the data must be further validated with a larger number of cases allowing a more robust statistical testing.

**Table 3:** Association between the expression of ZEB1 and other cellular markers. RNA transcripts were quantified in immunodepleted blood fractions by RT‐qPCR. Samples were considered positive if gene expression by 2^−ΔCq^ was equal to or higher than 2^−ΔCq^ + 1 SD as found in healthy donors. Association was calculated using Fisher's exact test. Significant *P*‐values are marked in boldface. Stem cell marker (SCM)

Variables	ZEB1		*P*‐value
	Positive	Negative	
CD24			
Positive	4	6	**0.054**
Negative	1	15	
CD44			
Positive	3	6	0.302
Negative	2	15	
CD133			
Positive	1	2	0.488
Negative	4	19	
All SCM			
Positive	5	10	**0.053**
Negative	0	11	
E‐Cadherin			
Positive	0	2	1.000
Negative	5	19	
Vimentin			
Positive	3	7	0.326
Negative	2	15	
Plastin3			
Positive	1	2	1.000
Negative	4	12	
LKB1			
Positive	0	5	0.283
Negative	7	15	

**Table 4:** Association between the expression of ZEB1 and clinicopathological characteristics. RNA transcripts were quantified in immunodepleted blood fractions by RT‐qPCR. Samples were considered positive if gene expression by 2^−ΔCq^ was equal to or higher than 2^−ΔCq^ + 1 SD as found in healthy donors. Association was calculated using Fisher's exact test. Significant *P*‐values are marked in boldface

Variables	ZEB1		*P*‐value
	positive	negative	
Tumor stage			
pT1	2	3	0.210
pT2‐3	3	19	
Nodal stage			
pN0	3	8	0.281
pN1‐3	2	11	
Histological grading			
G1‐2	4	6	**0.020**
G3	0	14	
ER/PR receptor status			
Positive	5	17	0.547
Negative	0	5	
HER2 status			
Positive	2	5	0.580
Negative	3	17	
Metastasis			
Bone			
Positive	2	5	1.000
Negative	3	17	
Visceral			
Positive	4	9	0.125
Negative	1	13	
Bone/visceral			
Positive	1	8	0.636
Negative	4	14	
Histological type			
Ductal	5	19	1.000
Lobular	0	3	
Therapy			
Chemo			
Positive	1	11	0.586
Negative	4	11	
Hormone			
Positive	4	17	1.000
Negative	1	5	
Radio			
Positive	2	14	0.340
Negative	3	7	
Herceptin			
Positive	2	5	0.184
Negative	3	17	

### 3.5 Colocalization of ZEB1, CD44, and LKB1 in patient‐derived CTCs demonstrates a heterogeneous cellular population

**Figure 4:**
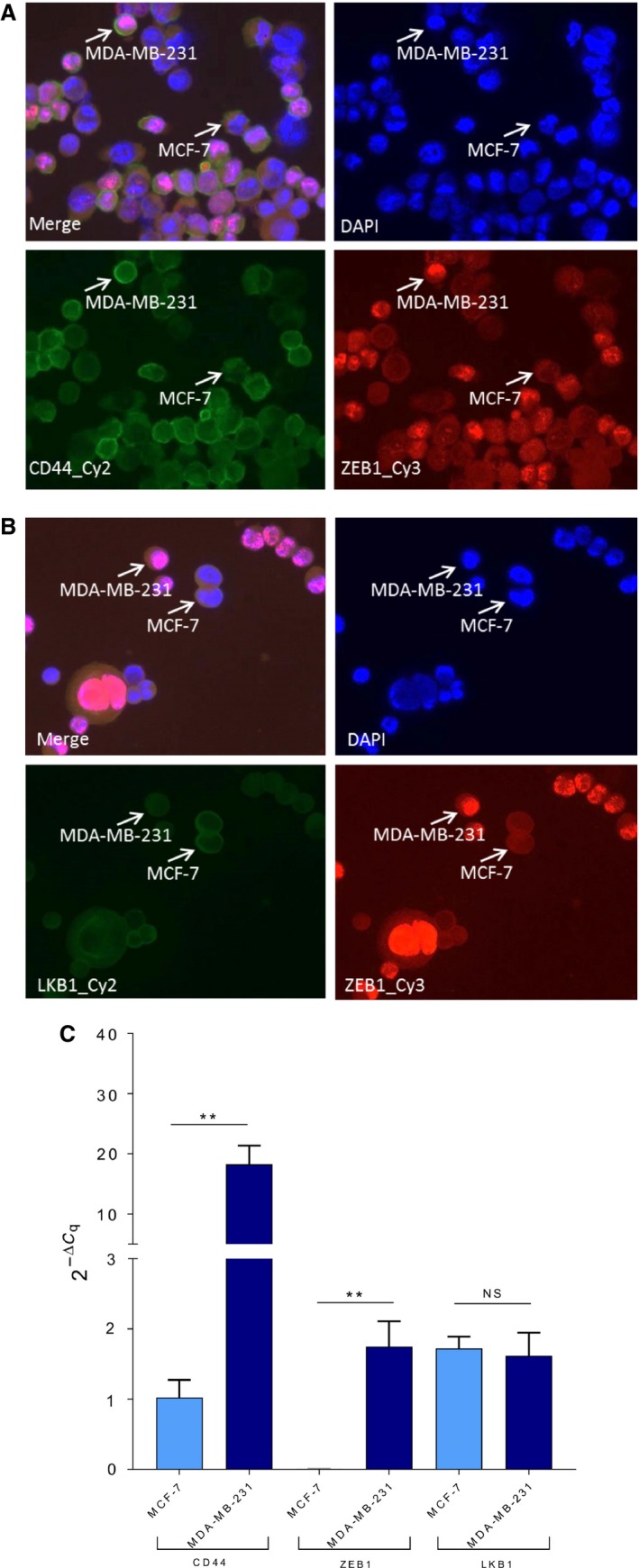
Double immunofluorescence staining of two different breast cancer cell lines that show an epithelial luminal phenotype (MCF‐7) or a mesenchymal, basal‐like phenotype (MDA‐MB‐231). (A) A mixed cell population, which contained both the phenotypes, was double‐stained with antibodies against ZEB1 or CD44 proteins. Samples were further incubated with CY2 (green)‐ or CY3 (red)‐labeled secondary antibodies. Nuclei were counterstained with 4′6‐diamidino‐2‐phenylindole (DAPI). (B) The mixed cellular population was double‐stained with antibodies against ZEB1 or LKB1. Samples were further incubated with CY2‐ or CY3‐labeled secondary antibodies. Nuclei were counterstained with DAPI. (C) Transcriptome analysis of ZEB1, LKB1, and CD44 in MCF‐7 and MDA‐MB‐231 breast cancer cell lines. In the same analysis, EpCAM, plastin 3, and vimentin were measured to confirm that the cell lines corresponded to the expected phenotype (data not shown). Each experiment was performed in triplicate and repeated six times. Data represent the mean values ±SD. Bars: standard deviation. Statistical analysis was performed using the Mann–Whitney *U*‐test (**P < 0.01; NS, not significant).

**Figure 5:**
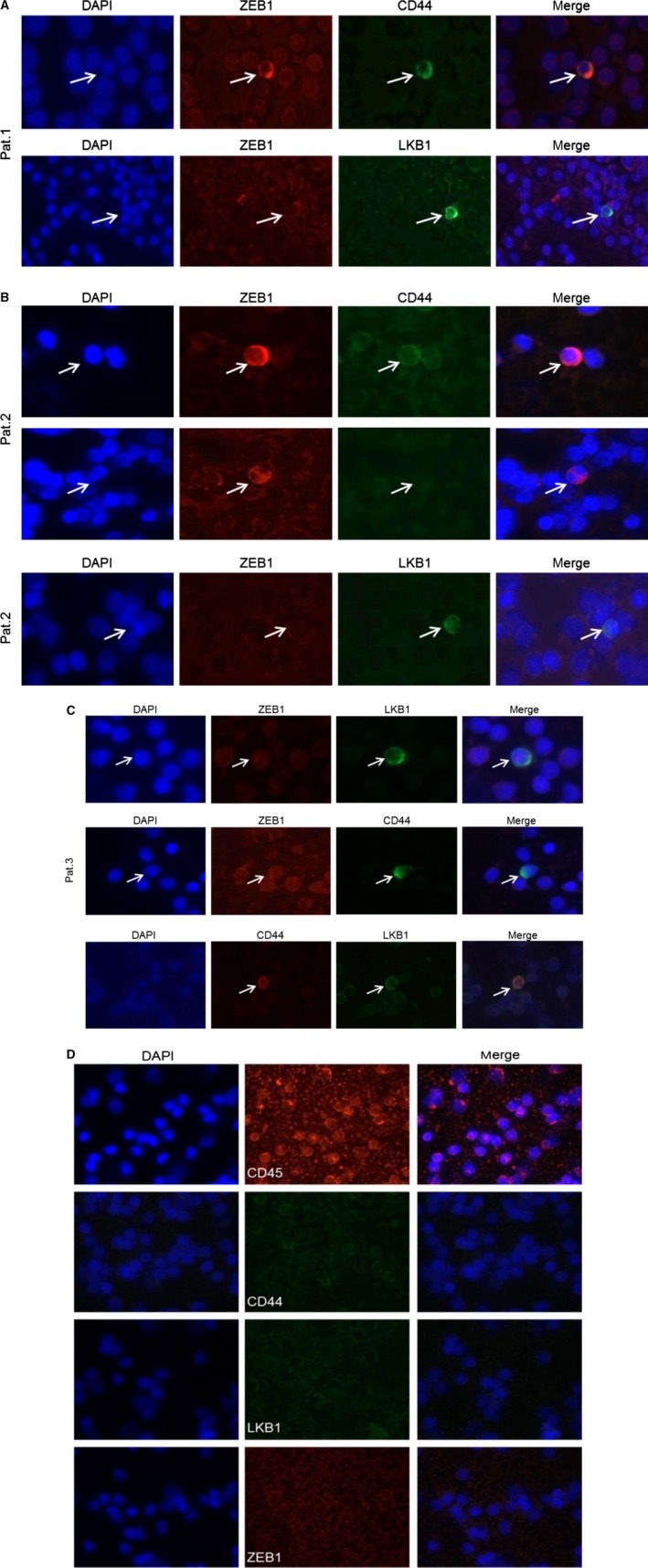
Identification of heterogeneous CTC subtypes that were detected by multifluorescence staining. Representative immunofluorescent images of single CTCs isolated from CS ^+^ patients (panel A), CS ^−^ patients (panel B), and patients showing enhanced expression of LKB1 and reduced expression of ZEB1 (panel C). White blood cells isolated from HDs were stained with the same antibodies to assess nonspecific signals. Samples were stained with antibodies against ZEB1, LKB1, and CD44. After incubation with the primary antibodies, samples were further incubated with CY2 (green)‐ or CY3 (red)‐labeled secondary antibodies. Nuclei were counterstained with DAPI. Nonspecific staining was not detected in the control samples (panel D).

As model system, we first assessed ZEB1, CD44, and LKB1 colocalization in two different breast cancer cell lines, the epithelial, luminal A subtype (MCF‐7) and the mesenchymal, metastatic type (MDA‐MB‐231), well representing the patients’ cohort in terms of primary tumor characteristics. Using immunofluorescence, we observed that MDA‐MB‐231 cells were simultaneously positive for CD44 and ZEB1 with ZEB1 showing a predominant nuclear localization (Fig. 4A). Conversely, MCF‐7 cells were negative for both markers or in the case of CD44, the signal was weak compared to MDA‐MB‐231 cells. The same cell lines were also costained for LKB1 and ZEB1. As expected, the ZEB1‐positive MDA‐MB‐231 cells were negative for LKB1, while LKB1 was mainly detected in the MCF‐7 cell cytoplasm (indicating an activated form) (Boudeau *et al*., 2003) where ZEB1 was not apparent (Fig. 4B). Accordingly, we analyzed and compared CD44, ZEB1, and LKB1 transcript levels between the two cell lines, and we confirmed that MDA‐MB‐231 cells expressed enhanced levels of CD44 and ZEB1 compared to MCF‐7 cells (Mann–Whitney test, *P *<* *0.05) (Fig. 4C). However, we did not detect a significant difference between the two cell lines relative to the expression of LKB1 (*P *=* *0.393), indicating rather a post‐transcriptional regulation of the protein. Next, we immunostained cytospins that were obtained from patient’ peripheral blood. In the CS^+^ patients, we distinguished two distinct cell populations displaying a ZEB1^+^/CD44^+^ or ZEB1^−^/LKB1^+^ phenotype (Fig. 5A). We were never able to detect cells that coexpressed LKB1 and ZEB1; this finding was consistent with the data from the transcriptome analysis. Conversely, ZEB1 and CD44 were always simultaneously detectable when CTCs were stained for both markers. In CS^−^ patients, we observed single CTCs with a ZEB1^+^/CD44^+^ or ZEB1^+^/CD44^−^ phenotype (Fig. 5B). We also detected cells with a ZEB1^−^/LKB1^+^, but never with a ZEB1^+^/LKB1^+^, phenotype. Unlike the cell lines, ZEB1 cellular localization in patients’ samples was cytoplasmic, which possibly indicated an inactive form of the protein. When we examined patients with enhanced LKB1 transcript levels and no ZEB1 expression, we observed CTCs with a ZEB1^−^/LKB1^+^ phenotype. However, these patients had nevertheless CTCs with a ZEB1^−^/CD44^+^ and LKB1^+^/CD44^+^ phenotype, suggesting that LKB1 was not interfering with CD44 expression (Fig. 5C). Blood samples collected from HDs were stained following the same protocol to assess nonspecific staining (Fig. 5D).

### 3.6 LKB1 is differentially expressed in epithelial and mesenchymal spheroids and is necessary for cell growth in suspension

**Figure 6:**
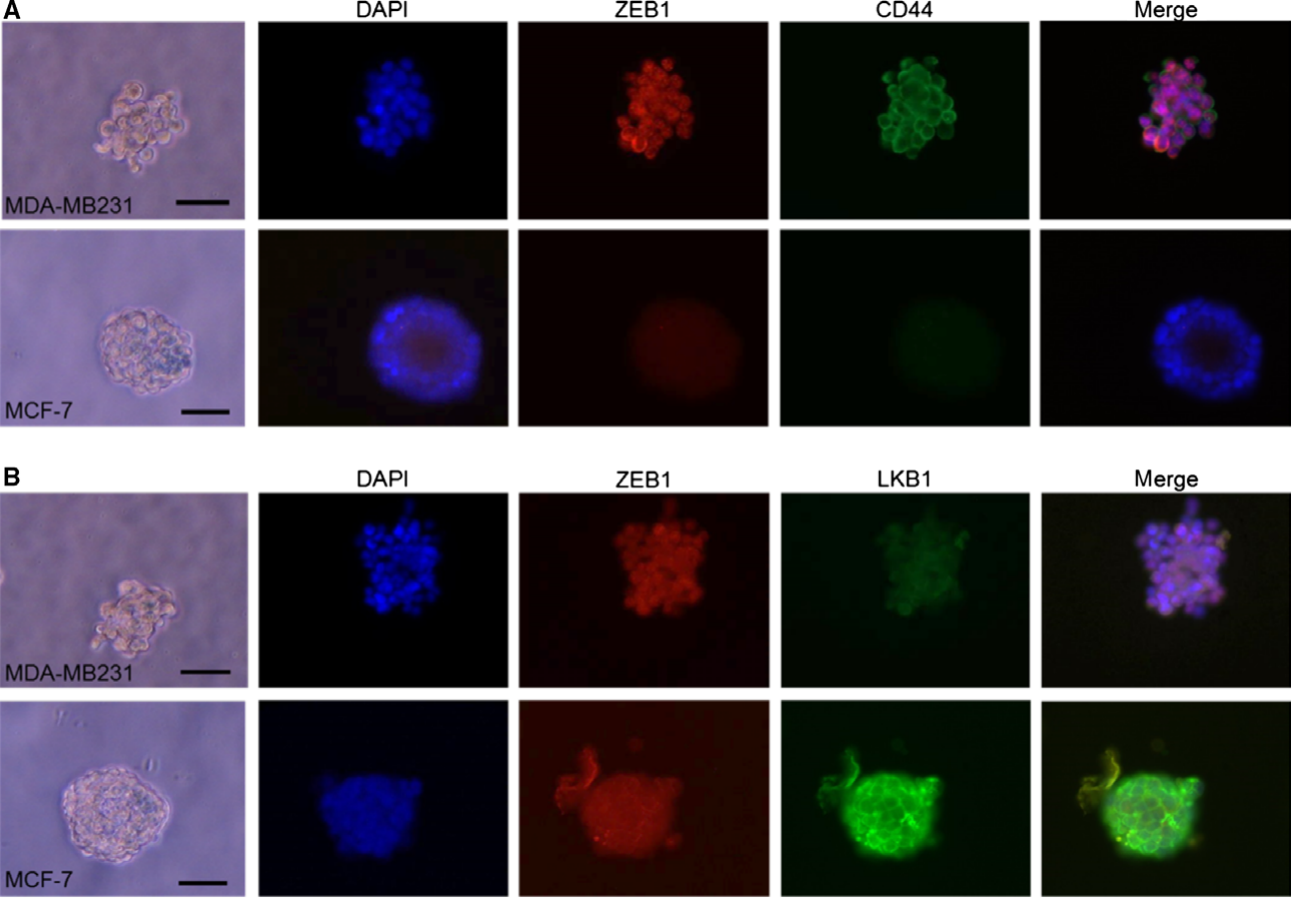
Tumor sphere formation by two different breast cancer cell lines. Epithelial, luminal‐like (MCF‐7) and mesenchymal, basal‐like (MDA‐MB‐231) breast cancer cells were seeded as single cells into ULA 6‐well plates and allowed to grow for 7 days in serum‐free medium. First‐generation tumor spheres (TS1) were transferred onto glass slides and stained with the ZEB1, LKB1, and CD44 antibodies, and nuclei were counterstained with 4′6‐diamidino‐2‐phenylindole (DAPI). As seen in the phase contrast images, TS1 from single MCF‐7 cells was round and compact, but TS1 from MDA‐MB‐231 consisted more of loose aggregates than proper spheres. (A) Double immunostaining of the TS1 from MDA‐MB‐231 (upper panel) or MCF‐7 (lower panel) single cells. The immunofluorescence analysis showed high levels of the ZEB1 and CD44 proteins in MDA‐MB‐231, but the two proteins were not detectable in MCF‐7. (B) Double immunostaining of the TS1 from MDA‐MB‐231 (upper panel) or MCF‐7 (lower panel) single cells only showed clear overexpression of LKB1 only in the epithelial, luminal‐like tumor cells; it was not detectable in the mesenchymal, basal‐type cells. ZEB1 was only detected in MDA‐MB‐231 cells. Scale bar: 20 μm.

**Figure 7:**
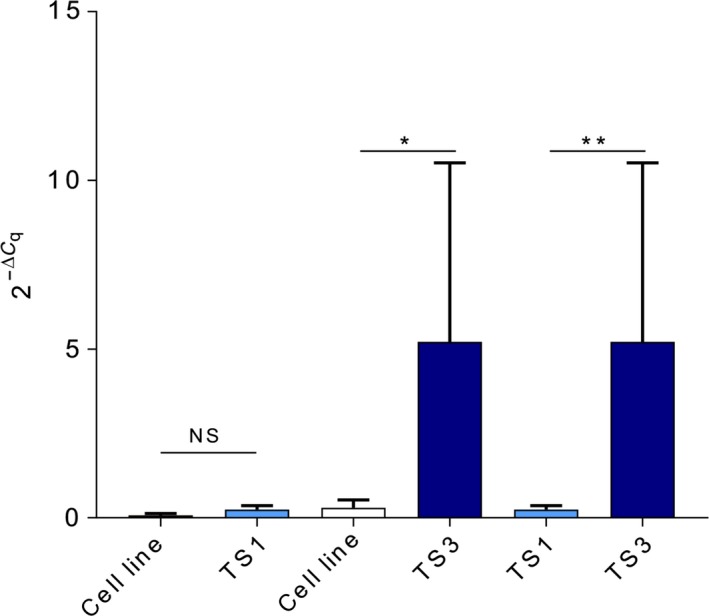
Transcriptome analysis of LKB1 in MDA‐MB‐231 cells. Cells were grown as a monolayer in standard conditions and collected as tumor spheres on days 7 (TS1) and 21 (TS3) days following seeding in serum‐free medium and in ULA dishes. Statistical analysis was performed using the Mann–Whitney *U*‐test (*P < 0.05, **P < 0.01, NS, not significant). Data represent the mean values ±SD. Bars: standard deviation (*n* = 3).

**Figure 8:**
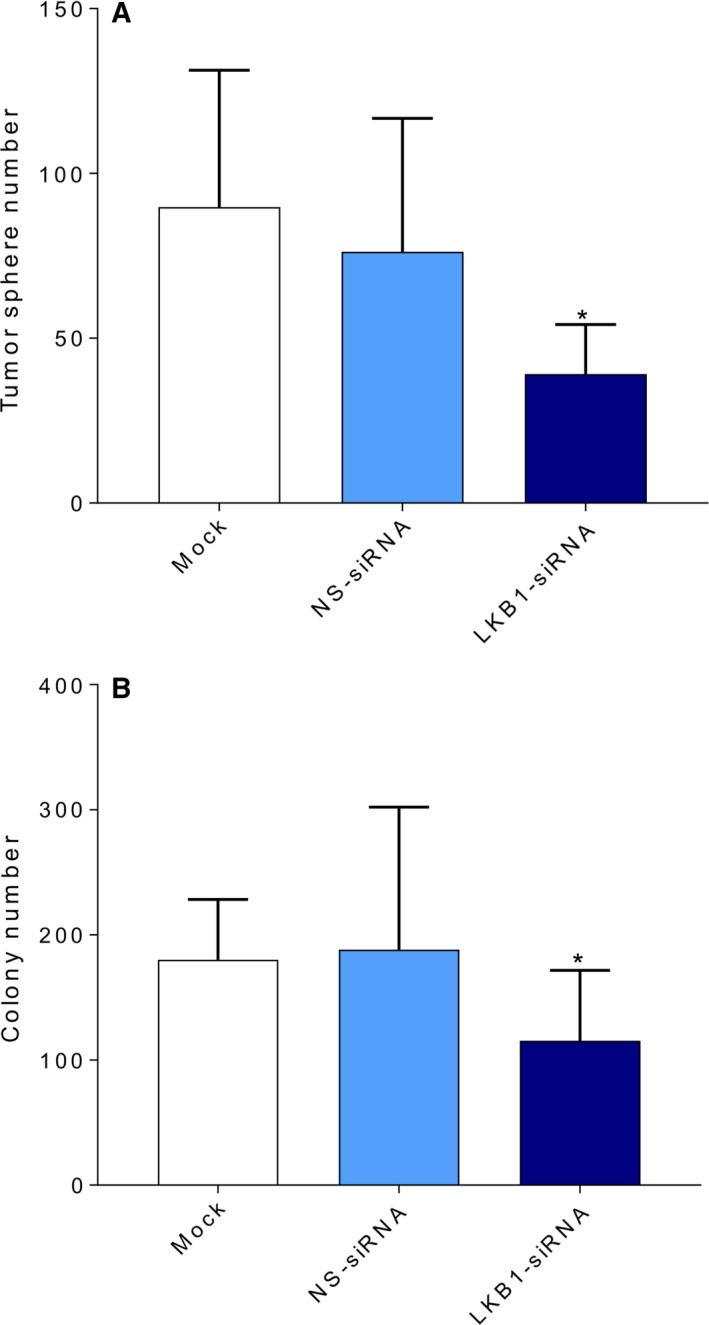
Targeted siRNA silencing of LKB1 in MCF‐7 cells. (A) LKB1‐siRNA‐ or nonspecific (NS)‐siRNA‐ transfected single MCF‐7 cells and mock cells were seeded into ULA 6‐well dishes (1 × 10^3^ cells per well) in serum‐free medium and allowed to grow as spheroids for 7 days. Statistical analysis was performed using the two‐tailed Friedman's test (*P < 0.05). Data represent the mean ± SD. Bars: standard deviation. (*n* = 5). (B) Clonogenic assays were performed after seeding LKB1‐siRNA or nonspecific (NS)‐siRNA‐transfected MCF‐7 cells and mock cells (1 × 10^3^ cells/10 cm dish) and allowing them in complete media for 14 days. Colonies with at least 50 cells were counted. Statistical analysis was performed using the two‐tailed Friedman's test (*P < 0.05). Data represent the mean ± SD. Bars: standard deviation. (*n* = 5)

To test whether LKB1 expression might be necessary to overcome anoikis in nonepithelial CTCs, we analyzed MCF‐7 and MDA‐MB‐231 cells in an *ex vivo* model system. Cells were seeded in ultra‐low adhesion (ULA) dishes as single cells and allowed to divide to form three subsequent generations of spheroids (TS1‐3). Similar to the cells that were cultured in as monolayers, MDA‐MB‐231 TS1 showed enhanced levels of ZEB1 and CD44 and low levels of LKB1; on the contrary, MCF‐7 TS1 showed low levels of ZEB1 and CD44 and high levels of LKB1 (Fig. 6A,B). The results were confirmed also by quantitative PCR (data not shown). Interestingly, when we analyzed the TS3 spheroids that were generated from MDA‐MB‐231 cells by qPCR, we observed a significant upregulation in LKB1 expression in the tumor spheres compared to the cells grown as monolayers (*P *=* *0.013) and to TS1 (*P *=* *0.002) (Fig. 7). The data generated with the mesenchymal cell line were confirmed in epithelial cells with a knock‐down model where LKB1 was silenced by siRNA. After transfection with specific and nonspecific siRNAs, MCF‐7 cells were either plated in ULA dishes in the absence of serum to test their ability to form spheroids, or heavily diluted in standard conditions to test their clonogenic capabilities. As expected, the MCF‐7 cells that were transfected with siRNA against LKB1 showed a reduced ability to generate colonies and to grow as spheroids in suspension, while proliferation of nonspecific siRNA‐transfected cells did not differ from the mock controls (Fig. 8). These results suggest that LKB1 might play a critical role in maintaining cell viability and proliferation ability in the absence of a substrate; therefore, upregulation of LKB1 in CTCs might represent one mechanism for overcoming anoikis when the cells lose contact with ECM.

## 4 Discussion

Epithelial cells tightly adhere to each other through different proteins such as cadherins (Pantel *et al*., 1998). However, epithelial cells possess high plasticity; they can detach from tissues and switch to a mesenchymal phenotype by reprogramming gene expression to undergo EMT. Carcinoma cells, by means of EMT, acquire invasive features and are able to leave the original tumor as CTCs. Once they reach a secondary site, CTCs undergo a reverse mesenchymal–epithelial transition (MET) to implant in a new tissue to begin a metastasis (Joosse *et al*., 2014; Tam and Weinberg, 2013). However, ECM detachment and circulation are events that severely hamper CTC survival; probably only a small fraction of CTCs, as single cells or small clusters, can overcome anoikis to acquire metastatic potential. In addition to its role as tumor suppressor, the protein kinase LKB1 possesses a pro‐oncogenic activity, where it functions as a potent sensor of low energy within cellular metabolism (Lee *et al*., 2015). It may be possible for LKB1 to rescue CTCs from metabolic stress and anoikis. LKB1/AMPK pathway activation in tumor cells might be associated with a block in EMT and the acquisition of a dormant cellular state, which are events that have been suggested for CTCs biogenesis and therapy resistance.

**Figure 9:**
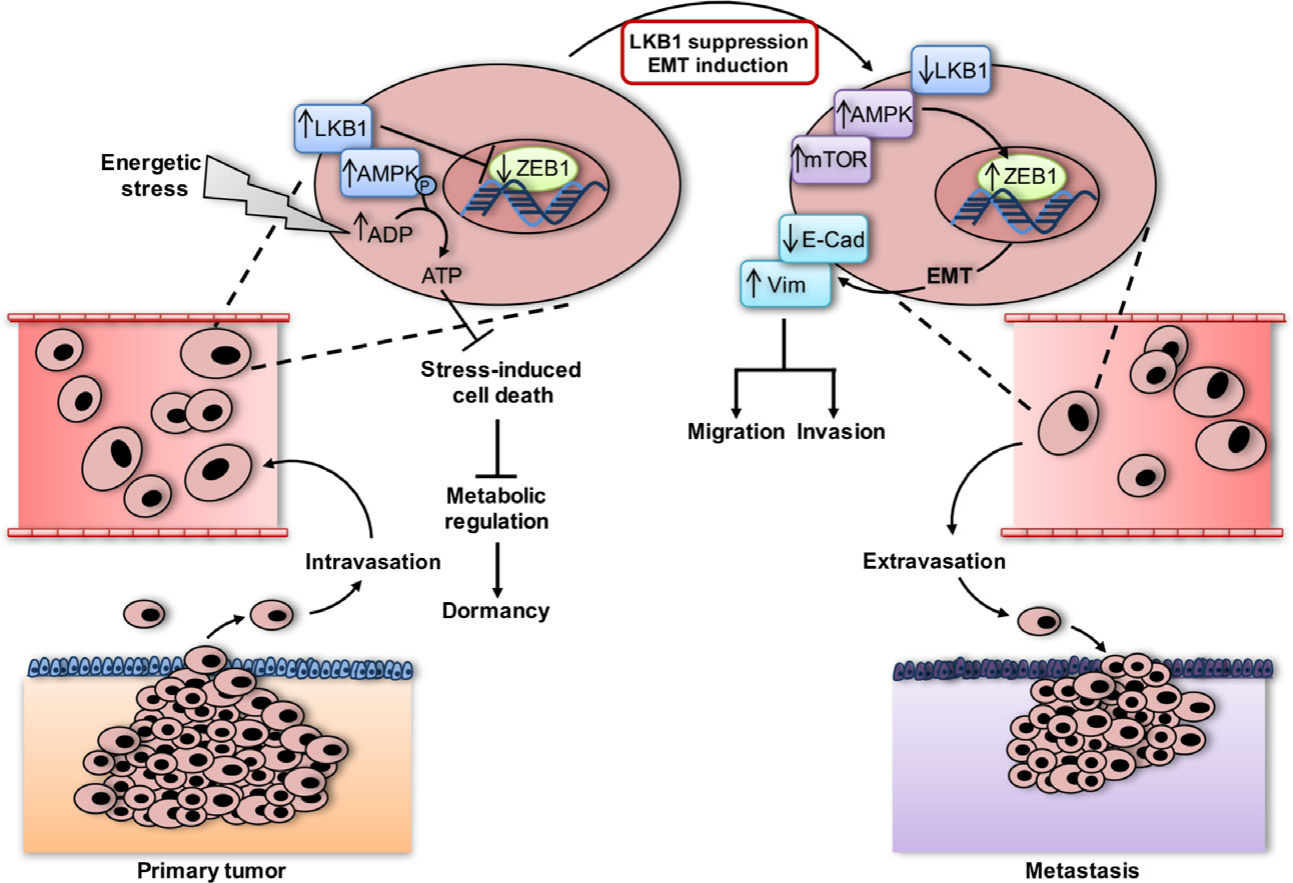
Proposed model for the sequential role of LKB1 and ZEB1 in EpCAM‐negative CTCs in metastatic breast cancer. Circulating tumor cells detach from the primary tumor and enter the blood stream through intravasation, a process that induces a strong energy stress, with consequent anoikis induction. To survive, CTCs must upregulate LKB1 to activate AMPK, phosphorylate ADP, and rescue the cells from metabolic stress. LKB1 also blocks ZEB1 expression, which inhibits its proliferative effect and pushes the cells into a dormant state. LKB1 expression is suppressed by different stimuli. AMPK activates ZEB1 through different signaling pathways (e.g., PI3K/AKT/mTOR), and ZEB1 drives CTCs through EMT, enhancing their migration and invasion properties. Once they reach a distant site, CTCs extravasate and undergo MET to restore the original epithelial phenotype and start metastasis.

In this study, we investigated a pool of CTCs with an EpCAM^−^ phenotype to determine whether LKB1 could trigger resistance to cellular death, possibly in the early phases of EMT. We observed that over 25% of the patients had enhanced levels of LKB1 and that LKB1‐positive samples were negative for ZEB1. LKB1 did not show a correlation with the CSC markers. However, ZEB1 did, which was consistent with studies that demonstrated the existence of a self‐enforcing CD44/ZEB1 feedback loop as a driving force of tumorigenesis and metastatic progression (Preca *et al*., 2015). Furthermore, we found that CS^−^ patients with enhanced levels of CD44 had a worse OS and PFS. LKB1 was not associated with the clinical outcome, suggesting that at least in CTCs LKB1/AMPK is more specifically linked to cell survival and potentially, to cell dormancy rather than aggressiveness and invasiveness. We can speculate that LKB1 plays a pro‐oncogenic role in CTCs, possibly during the earlier phases of tumor dissemination that is hampered by the activation of anoikis as a consequence of detachment from the primary tumor. LKB1 upregulation could be considered as the first step for intravasation, to help CTCs survive anoikis with a simultaneous block of ZEB1. Under specific signals, LKB1 expression could then be switched off, releasing the ZEB1 block to begin the metastatic process. The positive correlation between LKB1 upregulation and the presence of EpCAM^+^ CTCs supports the hypothesis that LKB1 expression is an event that occurs during the early phases of intravasation, when the cells have not completely transitioned from their epithelial phenotype. As expected, the immunofluorescence analysis of single CTCs that were isolated from patients showed a heterogeneous cellular population. We did not detect CTCs with simultaneous evident ZEB1 and LKB1 proteins, but we observed cells that were positive for both LKB1 and CD44, suggesting that CD44 upregulation may be independent from ZEB1. In support of the hypothesis that LKB1 upregulation might allow tumor cells to survive to anoikis once detached from the primary tumor, a spheroid model system showed that mesenchymal tumor cells, which are normally unable to grow in suspension, acquired the ability to expand and form spheres when they expressed higher level of LKB1; upregulation of ZEB1 and CD44 was irrelevant in this regard. LKB1 overexpression serves as the driving force to select mesenchymal CTCs that can overcome cell cycle arrest and anoikis (Shaw *et al*., 2004). This observation is supported by targeted knock‐down experiments in MCF‐7 breast cancer cells. LKB1 silencing showed that these cells were losing their viability in suspension or their clonogenic ability. It has already been shown that the LKB1/AMPK pathway rescues epithelial cells from anoikis once they detach from a surface (Ng *et al*., 2012) and that LKB1 plays an important role in mediating anoikis resistance in ovarian cancer (Peart *et al*., 2015). Accordingly, we propose that LKB1/AMPK signaling can support CTCs in breast cancer toward overcoming metabolic stress and a low energy supply, which are events that negatively affect intravasation. Furthermore, as proposed by Peart and collaborators, the lower metabolism and proliferation rates that are assured by a simultaneous ZEB1 block might be the reason why CTCs, as part of their biogenesis, can enter into a phase of dormancy (Aguirre‐Ghiso, 2007; Sosa *et al*., 2014). Downregulation of LKB1, with a concomitant ZEB1 block release, would then stimulate the switch from a quiescent to an active state with the consequent activation of EMT and acquisition of migratory and invasive potentials (Fig. 9). Because LKB1 might play a key role in CTC survival in the early phases of cellular dissemination, targeted gene expression downregulation or protein inhibition could be a novel and interesting treatment approach for early BC patients. LKB1‐negative cells are sensitive to drugs that alter the AMP/ATP ratio and induce cellular death, which presents new therapeutic approaches (Zhou *et al*., 2014). Additionally, targeting LKB1 in dormant CTCs may be an effective strategy for overcoming the limited power of chemotherapy in nonproliferating cells, which is a major problem in the treatment for minimal residual disease. Targeting CTCs to predispose them to metabolic stress‐induced anoikis may be therefore seen as a novel strategy for inhibiting and limiting metastasis during the early phases of tumor spread. Further studies including a larger patient's cohort will be necessary to validate the results obtained in this pilot study. It will be important to further optimize the isolation method to avoid as much as possible contaminations with other CD45^−^ cell types such as circulating endothelial cells (CECs) or nontumoral stem cells. Nevertheless, considering that CECs have been associated with tumor spreading and metastasis induction by supporting angiogenesis, it could be interesting to verify whether there indeed exists a synergy between CTCs and CECs in clinical outcome. If this would be the case, analyzing a pool of different cells would be not any longer a technical issue, rather an interesting study model.

## 5 Conclusions

The CTC analysis can be a powerful tool for performing liquid biopsies. However, CTC biogenesis and molecular characteristics must be clarified to provide necessary information for individualized therapy toward recurrence and metastasis prevention. LKB1 might be required for cell survival in the early phases of dissemination, when CTCs are characterized by a low metabolic, dormant state. Suppression of LKB1 and upregulation of ZEB1 would form the signal to awaken the cells to drive them into EMT. A larger patient population, a different time point analysis (ideally before surgery and at recurrence) and a more efficient way to separate CTCs from other CD45^−^ cell types will be necessary to validate these preliminary results and to support their clinical impact. However, our findings suggest a novel and better way to stratify patients and highlight LKB1 as a possible therapeutic target for metastasis‐initiating CTCs.

## Author contributions

EKT and MA‐F were responsible for the study conception, design, and data interpretation. MA‐F was responsible for the data analysis and manuscript writing. EKT, UA, and JK were responsible for the patient identification and sample collection. LM was responsible for the transcriptome data acquisition. BZ was responsible for the cell culture, targeted gene silencing, colony formation assays, tumor sphere preparation, and cellular immunofluorescence data acquisition. TWPF was responsible for the statistical analysis revision; and UA, HS, NH, SM, TWPF, WJ, and BR were responsible for the data interpretation and revision of the manuscript for intellectual content. All authors read and approved the final version of the manuscript.

## Supporting information


**Appendix S1.** Experimental material.
**Table S1.** List of the TaqMan^®^ Assays (Primer/Probe Set) used for the RT‐qPCR analysis.
**Table S2.** Association between the expression of LKB1 and other cellular markers or patients’ characteristics.
