# Supplementary material for: LKB1 pro‐oncogenic activity triggers cell survival in circulating tumor cells
Source: Mol Oncol. 2017 Sep 30;11(11):1508–26. doi: 10.1002/1878-0261.12111 (PMC5663996; doi:10.1002/1878-0261.12111)
Supplement: Supplementary file 1 — Appendix S1. Experimental material. Table S1. List of the TaqMan® Assays (Primer/Probe Set) used for the RT‐qPCR analysis. Table S2. Association between the expression of LKB1 and other cellular markers or patients’ characteristics. [file MOL2-11-1508-s001.docx]

Supplemental Experimental Material

Enrichment of EpCAM^-^ CTCs by immunodepletion

EpCAM^-^ CTCs were isolated from the remaining supernatants laid over 15 mL Ficoll-Paque PLUS (GE Healthcare Europe, Germany) and centrifuged at 400 x g for 30 min at RT. The buffy coat was collected in a fresh 50 mL conical tube filled with PBS/2mM EDTA buffer and further centrifuged at 300 x g for 10 min at RT. Remaining platelets were removed by two additional washing steps in 40-50 mL PBS/2mM EDTA buffer and centrifuged at 200 x g for 10 min at RT.

Isolation of total RNA

Total RNA isolation was performed using TRIzol LS Reagent (ThermoFisher Scientific, Germany) according to the manufacturer’s instructions with few modifications (see also Supplemental Experimental Material). Frozen cell pellets were thawed on ice and homogenized with 1 mL TRIzol LS Reagent. Lysates were incubated for 5 min at RT, vortexed vigorously after addition of 0.2 mL chloroform (Merck, Germany) followed by additional incubation at 10 min at room temperature. Samples were then centrifuged at 12.000 x g for 15 min at 4°C. The upper aqueous phase was aspirated carefully without disturbing the interphase, mixed with 500 µL 100% isopropanol (ThermoFisher Scientific, Germany) and stored overnight at -20°C. Samples were then centrifuged at 12.000 x g for 10 min at 4°C, supernatants were discarded and the RNA pellets were washed in 1 mL of 75% ethanol (ThermoFisher Scientific) followed by centrifugation at 12.000 x g for 8 min at 4°C. Pellets were air dried for 5 min, dissolved in 20 µL DNase/RNase-free water, incubated in a heat block at 55°C for 10 min and chilled on ice. To eliminate genomic DNA contamination samples were treated with Deoxyribonuclease I (DNase I) (ThermoFisher Scientific) according to the manufacturer’s instructions. Quality of RNA was checked by 2% agarose gel electrophoresis (SYBR Safe E-Gel 2%, ThermoFisher Scientific) and RNA yield was determined spectrophotometrically (NanoPhotometer, Implen, Germany).

Quantification of EpCAM^+^ CTC

Peripheral blood (7.5 mL) was mixed with dilution buffer provided within the CellSearch^TM^ Epithelial Cell Kit and centrifuged for 10 min at 800 x g at RT, samples were processed on the CellTracks^®^ AutoPrep^®^ (Janssen Diagnostics) system using the CellSearch^TM^ Epithelial Cell Kit (Janssen Diagnostics). After immuno-magnetic enrichment with an anti-EpCAM antibody, cells were further labeled with anti-cytokeratin (CK8,18,19–phycoerythrin) and -CD45 (CD45–allophycocyanin) fluorescent antibodies, while 4,6-diamidino-2-phenylindole dihydrochloride (DAPI) was used to detect the nuclei. The identification and enumeration of CTCs were performed using the CellTracks^®^ Analyzer^®^ II (Janssen Diagnostics). All positive samples were reviewed by two independent investigators.

Cytospins preparation

PBMCs were enriched using Ficoll-Paque PLUS (GE Healthcare, Germany) based on a density gradient centrifugation. Blood was diluted 1:4 with PBS/2mM EDTA, layered carefully onto 15 mL Ficoll-Paque PLUS media solution and was centrifuged at 400x g for 30 min at room temperature. All mononuclear cells from the interphase layer were collected, washed once in 40 mL PBS/2mM EDTA (centrifugation 300 x g for 10 min at RT) and twice in 30 mL PBS/2 mM EDTA (centrifugation 200 x g for 10 min at RT). PBMCs (1 x 10^6^ cells) resuspended in PBS/2mM EDTA were finally spun down at 150 x g for 5 min at RT on a glass slide (SuperFrost^®^ Plus; ThermoFisher Scientific). Cytospins were dried for 12-24 hours at RT and then stored at -80°C.

Table S1

| Gene name (Identification number) | Efficiency calculated from slope (%) | r^2^ values |
| --- | --- | --- |
| Reference gene: |  |  |
| B2M (Hs00984230_m1) | 102.1 | 0.999 |
| ß-Actin (Hs99999903_m1) | 86.6 | 0.998 |
| GAPDH (Hs99999905_m1) | 104.8 | 0.999 |
| GUSB (Hs00939627_m1) | 103.2 | 0.999 |
| HPRT1 (Hs00939627_m1) | 100.3 | 0.998 |
| PPIA (Hs04194521_s1) | 101.0 | 0.998 |
| TBP (Hs00427620_m1) | 91.6 | 0.999 |
| TUBB (Hs00742828_s1) | 91.8 | 0.998 |
| UBC (Hs01871556_s1) | 93.6 | 0.999 |
| 18S rRNA (Hs99999901_s1) | 91.2 | 0.999 |
| Target gene:  CD24 (Hs02379687_s1) | 99.2 | 0.998 |
| CD44 (Hs01075861_1) | 92.7 | 0.999 |
| CD133 (Hs01009250_m1) | 82.9 | 0.990 |
| E-Cadherin (Hs01023894_m1) | 97.4 | 0.998 |
| N-Cadherin (Hs00362037_m1) | 103.6 | 0.999 |
| EpCAM (Hs00901885_m1) | 90.0 | 0.992 |
| LKB1 (Hs00176092_m1) | 85.7 | 0.999 |
| SLUG (Hs00161904_m1) | 98.4 | 0.999 |
| SNAIL1 (Hs00195591_m1) | 91.9 | 0.991 |
| TWIST1 (Hs00361186_m1) | 91.2 | 0.988 |
| ZEB1 (Hs01566408_m1) | 94.4 | 0.999 |
| Vimentin (Hs00185584_m1) | 94.0 | 0.999 |
| Plastin 3 (Hs009583554_m1) | 101.2 | 0.999 |

Table S2

| Variable |  | LKB1 | | *p -* value |
| --- | --- | --- | --- | --- |
|  |  | positive | negative |  |
| CD24 |  |  |  |  |
|  | positive | 2 | 8 | 0.678 |
|  | negative | 5 | 12 |  |
| CD44 |  |  |  |  |
|  | positive | 2 | 7 | 1.000 |
|  | negative | 5 | 13 |  |
| E-Cadherin |  |  |  |  |
|  | positive | 0 | 2 | 1.000 |
|  | negative | 7 | 18 |  |
| Vimentin |  |  |  |  |
|  | positive | 1 | 9 | 0.204 |
|  | negative | 6 | 11 |  |
| Tumor stage |  |  |  |  |
|  | pT1 | 0 | 4 | 0.545 |
|  | pT2-3 | 7 | 16 |  |
| Nodal stage |  |  |  |  |
|  | pN0 | 4 | 6 | 0.650 |
|  | pN1-3 | 3 | 10 |  |
| Histological grading |  |  |  |  |
|  | G1-2 | 1 | 9 | 0.171 |
|  | G3 | 6 | 8 |  |
| ER/PR receptor status |  |  |  |  |
|  | positive | 5 | 17 | 0.579 |
|  | negative | 2 | 3 |  |
| HER2 status |  |  |  |  |
|  | positive | 0 | 6 | 0.145 |
|  | negative | 7 | 13 |  |
| Metastasis |  |  |  |  |
| Bone | positive | 1 | 8 | 0.363 |
|  | negative | 6 | 12 |  |
| Visceral | positive | 3 | 8 | 1.000 |
|  | negative | 4 | 12 |  |
| bone/visceral | positive | 3 | 6 | 0.652 |
|  | negative | 4 | 14 |  |
| Histological type |  |  |  |  |
|  | NST | 7 | 17 | 0.545 |
|  | Lobular | 0 | 3 |  |
| Therapy |  |  |  |  |
| Chemo T | positive | 3 | 8 | 0.586 |
|  | negative | 1 | 9 |  |
| Hormon T | positive | 5 | 16 | 0.633 |
|  | negative | 2 | 4 |  |
| RadioT | positive | 4 | 8 | 0.668 |
|  | negative | 1 | 8 |  |
| Herceptin | positive | 0 | 6 | 0.262 |
|  | negative | 5 | 10 |  |
